# A Review of 3D-Printing of Microneedles

**DOI:** 10.3390/pharmaceutics14122693

**Published:** 2022-12-01

**Authors:** Michael Olowe, Santosh Kumar Parupelli, Salil Desai

**Affiliations:** 1 Department of Industrial and Systems Engineering, North Carolina Agricultural and Technical State University, Greensboro, NC 27411, USA; 2 Center of Excellence in Product Design and Advanced Manufacturing, North Carolina Agricultural and Technical State University, Greensboro, NC 27411, USA

**Keywords:** 3D-printing, artificial intelligence, drug delivery, FDA regulations, microneedles

## Abstract

Microneedles are micron-sized devices that are used for the transdermal administration of a wide range of active pharmaceutics substances with minimally invasive pain. In the past decade, various additive manufacturing technologies have been used for the fabrication of microneedles; however, they have limitations due to material compatibility and bioavailability and are time-consuming and expensive processes. Additive manufacturing (AM), which is popularly known as 3D-printing, is an innovative technology that builds three-dimensional solid objects (3D). This article provides a comprehensive review of the different 3D-printing technologies that have the potential to revolutionize the manufacturing of microneedles. The application of 3D-printed microneedles in various fields, such as drug delivery, vaccine delivery, cosmetics, therapy, tissue engineering, and diagnostics, are presented. This review also enumerates the challenges that are posed by the 3D-printing technologies, including the manufacturing cost, which limits its viability for large-scale production, the compatibility of the microneedle-based materials with human cells, and concerns around the efficient administration of large dosages of loaded microneedles. Furthermore, the optimization of microneedle design parameters and features for the best printing outcomes is of paramount interest. The Food and Drug Administration (FDA) regulatory guidelines relating to the safe use of microneedle devices are outlined. Finally, this review delineates the implementation of futuristic technologies, such as artificial intelligence algorithms, for 3D-printed microneedles and 4D-printing capabilities.

## 1. Introduction

Additive manufacturing (AM) is a technology that is evolving very rapidly and currently has applications in advanced manufacturing and in our day-to-day lives [1,2,3,4,5,6]. It is also referred to as three-dimensional (3D)-printing, layered manufacturing, rapid prototyping, or solid free-form fabrication. This manufacturing approach uses computer-aided design (CAD) files to build three-dimensional objects for applications in the biomedical, health care, manufacturing, fashion, food industry, military, automotive, and aerospace sectors. AM was first developed in the 1980s, when Chuck Hull invented the first form of 3D-printer technology called stereolithography. He was the first to build an AM method that utilized CAD files in order to build 3D objects using rapid prototyping systems. AM technologies build objects from the bottom up by adding material one cross-sectional layer at a time [7,8]. The layers are built in the X-Y direction and are consolidated in order to generate the third dimension, which is the z-dimension. AM gives engineers the flexibility to collaborate and design customizable, complex products from any location in a timely fashion, which in turn breaks the barriers of localized engineering or manufacturing [4,9,10,11,12]. Additive manufacturing mainly consists of the following five basic steps to build 3D objects:

1. A computerized 3D solid model is developed;
2. It is converted into a standard AM file format, such as the standard tessellation language format (STL) [13];
3. The STL file is sent to a 3D printer where it is modified, e.g., changing the position and orientation of the part or scaling the part;
4. The part is built layer-by-layer on the 3D-printing machine;
5. The cleaning, finishing, and post-processing of the printed parts are conducted.

The advantages and disadvantages of AM processes are illustrated in Table 1. AM processes build three-dimensional objects in a layer-by-layer fashion, as discussed earlier, and can be utilized to rapidly develop 3D structures with complicated designs that are based on a computer-aided design (CAD) model. AM processes are compatible with various types of materials, such as metals, polymers, biomaterials, ceramics, and composites [14,15,16,17,18,19]. The capability to utilize biomaterials in AM processes enables the fabrication of a wide range of 3D structures for clinical and point-of-care applications, including tissue engineering, stem cell research, wound healing, organ-on-chip technology, cancer research assays, and cosmetics [20,21,22,23,24,25].

AM manufacturing processes enable the fabrication of macro- and micro-scale 3D structures for patients with special requirements and materials. AM is a promising technique that can be used for the fabrication of customizable, complex, and cost-effective microneedle arrays. AM techniques such as stereolithography (SLA), selective laser sintering (SLS), digital light processing (DLP), fused deposition modeling (FDM), two-photon polymerization (2PP), and continuous liquid interface production (CLIP) can manufacture microneedle arrays [26,27,28,29]. Generally, the microelectromechanical fabrication techniques such as molding, chemical wet etching, dry etching, direct laser micromachining, ultraviolet (UV) lithography, and micro milling are used for the fabrication of microneedle-based devices. The limitations of the traditional manufacturing techniques include advanced manufacturing facilities, time-consuming processes, expensive specialized equipment, limited customizability, and a lack of flexibility over specific parameters, such as array size, height, and aspect ratio [30]. These above-mentioned limitations can be addressed with the AM processes. Each AM process has a particular set of tradeoffs in terms of biocompatibility, design structure, resolution, cost-effectiveness, material type, and particular application. Other 3D-printing technologies include direct-write inkjet methods that use a combination of different materials and inks [31,32,33,34,35,36,37,38]. The emerging applications of 3D-printed microneedles include healthcare systems, tissue engineering, biomedical engineering, and healthcare systems. These applications specifically include drug and vaccine delivery, cosmetics, therapy, diagnosis, sample extraction, and stem cell research.

There are numerous review papers that have been published in various peer-reviewed journals about microneedles, but to our knowledge, there are no review articles that mainly focus on the additive manufacturing of microneedles for drug delivery applications with detailed FDA guidelines. This review article describes the progression of and the most recent breakthroughs in 3D-printed microneedles in the last decade. The classification of microneedles based on the application type is discussed. A comprehensive understanding of the various 3D-printing processes that are used for the fabrication of microneedles is also mentioned, along with the challenges and the applications. The emerging applications of 3D-printed microneedles in healthcare systems and biomedical engineering are reviewed. Detailed Food and Drug Administration (FDA) guidelines for the fabrication of microneedles are presented. Finally, the future prospects of 3D-printed microneedles are delineated in order to advance their application in various fields.

### 1.1. Microneedles

Microneedles (MNs) are minimally invasive, tiny needle devices that can be fabricated from a variety of materials, such as biomaterials, metals, polymers, ceramics, and composites [39,40,41], which are designed to penetrate the skin’s stratum corneum layer for various applications. The aim of microneedles is the delivery of bioactive materials, vaccines, and pharmaceutical agents, and the collection of bio-signals and substances from the body with minimal invasiveness. The administration of drugs through the gastrointestinal passage has not been the most efficient due to the poor absorption of orally ingested drugs and the pharmacokinetic activities of the body, which leaves only a fraction of the drug to achieve its intended therapeutic effect. Patients’ compliance with the conventional use of hypodermic needles has dropped significantly over the years due to the pain, anxiety, and discomfort that accompany their usage. A more appealing approach that offers the possibility of controlled release at the expense of the time of administration is transdermal drug delivery (TDD) using a microneedle patch. However, TDD is severely limited by the inability of most single drug particles to cross the skin at therapeutic rates due to the great barrier that is imposed by the skin’s stratum corneum layer [42]. In order to increase the skin’s permeability, different approaches have been investigated, including, but not limited to, chemical lipid enhancers, electric field employing iontophoresis, and electroporation to pressure waves that are generated by ultrasound or photoacoustic effects [43]. An alternative approach involves creating a pathway of micron-scale needles that serves to create microscopic holes on the outermost layer, called the stratum corneum, by inserting microneedles that are made of silicon, metal, or polymeric material. Microneedle arrays are promising devices in transdermal drug delivery applications. 

**Figure 1:**
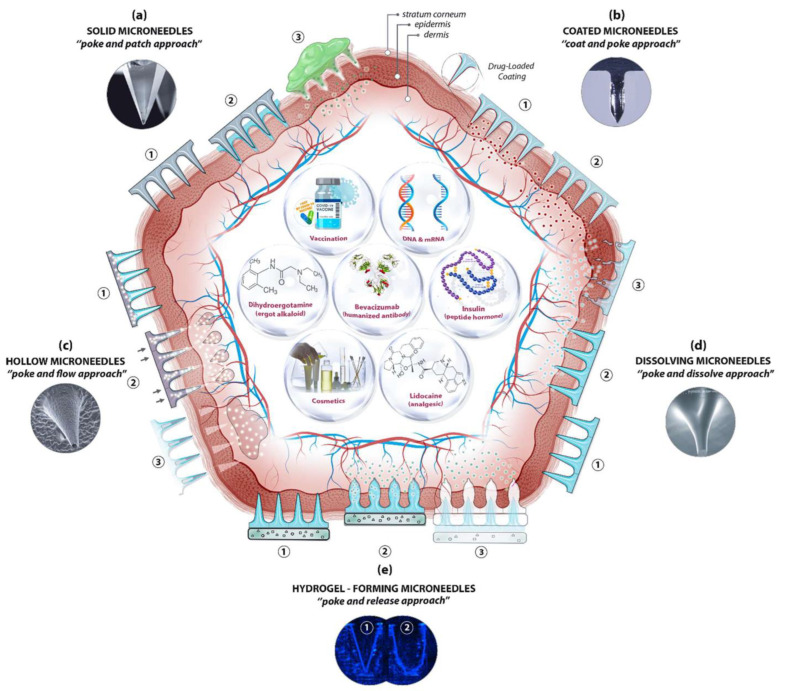
A schematic diagram of microneedle-based drug delivery approaches with a cross-section of the upper layer of the skin. The approaches are (**a**) solid microneedles, (**b**) coated microneedles, (**c**) hollow microneedles, (**d**) dissolving microneedles, and (**e**) hydrogel-forming microneedles. The step-by-step process of each delivery approach is numbered from 1 to 3 [46].

Microneedles are designed to be able to create an easier passage to the rich blood supply in the lower dermal layers, allowing an easy, pain-free delivery of a wide range of medicines across the skin [44]. The advantages of microneedles include painless administration, faster healing, ease of administration, and more control over the rate of drug delivery. Microneedle patches are categorized into five types, as shown in Figure 1, which include the following: solid microneedles, coated microneedles, dissolvable microneedles, hollow microneedles, and hydrogel-based microneedles. Each microneedle type has particular fabrication procedures and application areas. The first report of the term “microneedle” dates back to 1921 (Chambers, 1921), as a means of the micro-dissection of echinoderm eggs [45]. 

The microneedle concept started with using large needles, until it evolved over the years to the current micro-sized needles. In 1905, a German dermatologist used a motor-powered dental brush to treat skin ailments [47]. In the 1970s, Gerstel et al. introduced the microneedle concept, however, this concept was not demonstrated experimentally until the 1990s [48]. In 1998, Henry et al. were the first to propose a microneedle to be used for transdermal drug delivery [49]. The microneedle array in this study was fabricated using silicon as the material, with etching and photolithography as the manufacturing techniques. Initially, the purpose of the microneedle was to increase the skin’s permeability by using a solid microneedle. Another purpose was to fabricate hollow microneedles with advanced functionality compared to the ordinary hypodermic needles [50]. Eventually, this concept was extended to different applications, such as drug delivery, vaccine delivery, therapeutics, diagnostics, and cosmetic applications. A variety of materials, such as silicon, stainless steel, sugar, and polymers, have been used in order to fabricate solid microneedles, coated microneedles, hollow microneedles, or dissolvable microneedles [51]. Silicon was the first proposed material for fabricating a solid microneedle [49]; however, many other materials were studied in order to manufacture microneedles, such as stainless steel [52], ceramic [53], glass [54], and polymer [55]. Various types of manufacturing methods and techniques have been utilized over the years to fabricate specific and customized microneedle arrays. These manufacturing methods include, but are not limited to, lithography [56,57], micro milling, mold-based techniques, injection molding [58], laser ablation [59,60], an elasto-capillarity-driven self-assembly mechanism, and additive manufacturing [27,61]. Many of these conventional fabrication methods have some limitations, such as cost-efficiency, requiring manual steps, requiring sophisticated equipment, and being labor-intensive. Hence, accessible and cost-efficient technologies, such as additive manufacturing, are needed in order to produce microneedles. A summary of the fabrication techniques for the different types of microneedles is illustrated in Table 2.

### 1.2. Classification of Microneedles

**Figure 2:**
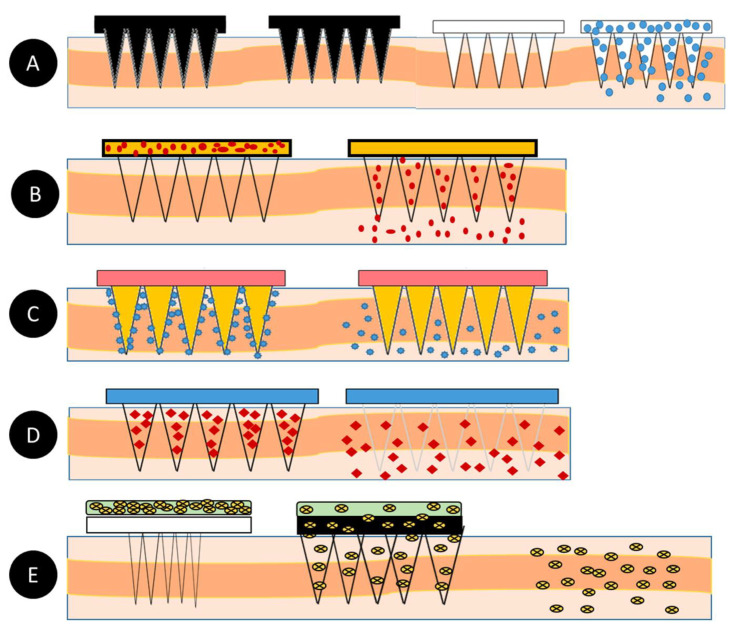
(**A**)—Solid microneedles; (**B**)—Hollow microneedles; (**C**)—Coated microneedles; (**D**)—Dissolving microneedles; (**E**)—Hydrogel-forming microneedles [40].

As mentioned earlier, microneedles are classified into five types, as shown in Figure 2, which include solid microneedles, hollow microneedles, coated microneedles, hydrogel-forming microneedles, and dissolving microneedles. The details about the material composition of microneedles and their benefits were summarized in Table 3.

#### 1.2.1. Solid Microneedles

Solid microneedles can be used to create microscopic holes in the skin through which molecules and therapeutic agents can be easily transported. This type of microneedle structure is designed to penetrate the stratum corneum in order to enhance the drug delivery to the dermis in order to improve the bioavailability and the kinetic transport across the skin [51]. The first microneedle arrays that were reported in the literature were etched into a silicon wafer and were developed for intracellular delivery in vitro by Hashmi et al. [63]. In comparison with hollow microneedles, solid microneedles have better mechanical properties [64,65], and the sharper tips are easier to manufacture. Solid microneedles are mostly used for pre-treating the skin by forming pores [66,67,68]. The pointed tips of the needles penetrate the skin, creating channels of micron size through which the drug directly enters the skin layers via the application of a drug patch, thus increasing the permeation [62].

#### 1.2.2. Hollow Microneedles

Hollow microneedles can accommodate a large dose of the drug dispersion or solution, as higher amounts can fill up the empty space inside of the needle. They are mostly used for high molecular weight compounds, such as proteins and vaccines [69]. Unlike solid microneedles, hollow microneedles are active drug delivery systems that form a channel for the efficient diffusion of drugs into the dermal layer based on a non-pressurized drug reservoir [51]. Hollow microneedles can be designed to allow for the modulation of the flow, the pressure, and the drug release rate [51,70]. The microneedle aspect ratio can be controlled for a rapid release, a slow infusion, or a time-varying delivery rate [71]. Mishra et al. developed hollow microneedles that were aligned on the silicon substrate with a length of 500–600 μm and a 100 μm outer diameter that achieved a flow rate of 0.93 μL s^−1^ at a pressure differential of 2 KPa [72,73]. Hollow microneedles have the disadvantage of clogging and leakage during the injection procedure. They are also relatively weaker and require intensive care in terms of the needle design and the insertion methods [74].

#### 1.2.3. Coated Microneedles

A coated microneedle comprises a sharp, solid-core microneedle structure on which a solid film containing the active compound and water-soluble inactive excipients are coated [75]. A coated microneedle can deliver proteins and DNA in a minimally invasive manner [76]. An advantage of a coated microneedle is the rapid delivery of the drug to the skin; however, the remnant drug at the tip of the needle might infect other patients [76]. In contrast to the dissolvable microneedle, whose mechanical properties can change when the encapsulated drug fraction is altered or when a different drug is dispersed in its matrix, the mechanical properties of a solid microneedle are not impacted when a different drug is coated on its surface [75].

#### 1.2.4. Dissolving Microneedles

Dissolving microneedles are made from biodegradable polymers [77,78]. They are designed to encapsulate the drug agents and to control their release upon the degradation and dissolution of the polymers into the skin. This type of microneedle differs from the others in that it does not have to be removed after its insertion. The bio-acceptability and the dissolution of the polymer inside the skin make it one of the best choices for long-term therapy, with improved patient compliance [79]. Water-soluble materials are the most suitable for manufacturing dissolvable microneedles. In addition, the micro-molding method is one of the most appropriate techniques for producing dissolvable microneedles. The micro-molding procedure involves filling the microneedle mold with a concentrated polymer solution and drying or filling it with melted polymer and allowing it to solidify. One of the biggest challenges of using dissolvable microneedles is that there can be a delay in the dissolution and complete insertion is impracticable [62].

#### 1.2.5. Hydrogel-Forming Microneedles

Hydrogel-forming microneedles are usually made up of swelling materials or aqueous polymer gels. This type of microneedle works by absorbing the interstitial fluid when it is applied to the skin and swells, resulting in the formation of channels between the capillary circulation and the drug patch. Upon the application and the swelling of these microneedles, they behave as a rate-controlling membrane. Hydrogel-forming microneedles can be easily sterilized and removed from the skin where they are applied [79,80,81]. Hydrogel-forming microneedles can also be fabricated using cross-linked polymers. Hydrogel-forming microneedles help to improve the permeation and bioavailability when they are used in transdermal drug delivery [50,82].

## 2. Additive Manufacturing Techniques

**Figure 3:**
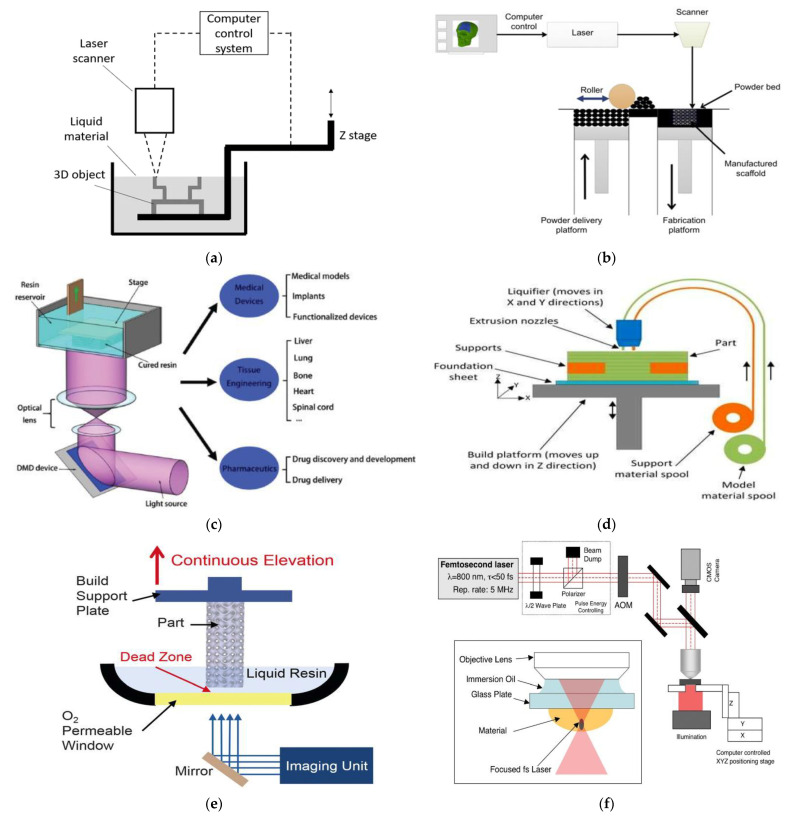
Schematics of the AM techniques (**a**) First-generation stereolithography [85], (**b**) Selective laser sintering [103], (**c**) Digital light processing [90], (**d**) Fused Deposition Modeling [104], (**e**) Continuous liquid interface production [92], and (**f**) Two-photon polymerization process [105].

Additive manufacturing is popularly known as 3D-printing. The different types of 3D-printing processes (Figure 3) that can be utilized for the fabrication of microneedles include stereolithography (SLA), selective laser sintering (SLS), digital light processing (DLP), fused deposition modeling (FDM), continuous liquid interface production (CLIP), and two-photon polymerization (2PP).

### 2.1. Stereolithography (SLA)

The first breakthrough in stereolithography happened in the 1970s [83,84]. In this method, three-dimensional objects are built layer-by-layer by curing photosensitive materials with ultraviolet radiation. Over the years, different approaches have been developed for stereolithography systems, which are classified into four generations [85]. The first generation involved scanning a laser beam over a liquid material, as developed by Hull; this method produced three-dimensional structures with a low efficiency. In order to overcome this challenge of low efficiency, the second generation of stereolithography (called projection stereolithography) was developed, which had the ability to cure each layer concurrently using photomasks. The third generation of stereolithography was developed in 2015, which was able to print the parts much faster than the earlier versions could. A volumetric stereolithography system has also been reported in the literature by Shusteff, M. et al. [86]. This method can produce 3D objects with a unit of complex aperiodic 3D volumes within seconds. The other types of stereolithographic systems that are available are thermal and color stereolithography [87]. The stereolithography process is fast and can produce almost any design, however, it can be very expensive.

### 2.2. Selective Laser Sintering (SLS)

SLS is the first commercialized form of the powder bed fusion AM technique that was developed by four scientists at the University of Texas, Austin (Gong H, Rafi K, Gu H, and Starr B) [88,89]. This method was first developed for plastic samples, using pointwise laser scanning technology, although it has now been extended for use with metal and ceramics, with the incorporation of additional thermal sources. The powder material (which is made of polymer or ceramics) is fed through the feed cartridges into the powder bed. The powder material is preheated using infra-red heaters, which serve to maintain the temperatures of the parts that are to be formed and to reduce the requirement of the C0_2_ laser. The roller on the build platform spreads the powder material, having a thickness of fewer than 100 microns across the built area, and the laser beam binds the material together. This process is repeated in a layer-by-layer fashion from the bottom up, until the whole part is built.

### 2.3. Digital Light Processing (DLP)

In this method, the 3D-printed objects are formed using projection light to polymerize the materials in order to obtain the desired shape and configuration. The technology has significant advantages in the printing resolution, efficiency, and working conditions over the conventional three-dimensional printing methods, such as the extrusion-based and ink-jet-based 3D-printing methods [90]. The DLP method, as shown in Figure 3c, uses a digital micromirror device that is made of micro-scale mirrors that rotate continuously to control the light path and the light rays onto the photosensitive resins during the operation. There are up to two million mirrors on the ordinary rays of light. The printing resolution of the DLP printers is relatively high and is usually at the micron-scale level [90]. Unlike laser-printing technology, the DLP printer focuses the light on a spot, thereby ensuring that the entire layer is printed at once and the printing is carried out in a swift manner.

### 2.4. Fused Deposition Modeling (FDM)

FDM is an extrusion-based technology that was developed in the late 1980s. In this AM technique, the feedstock filament is fed from a reel into the printer nozzle head and its extrusion is controlled by a motor. In this process, the modeling and the support materials are used for producing the finished three-dimensional objects and as a temporary support, respectively [91]. The materials that are used in this process are made from ABS, PLA, PS, PC, PEI, and nylon. The FDM method is one of the most economical additive manufacturing techniques, however, it has limitations such as speed and layer thickness accuracy [91].

### 2.5. Continuous Liquid Interface Production (CLIP)

Continuous liquid interface production is a type of photopolymerization that can achieve build speeds that are much faster than the conventional layer-by-layer stereolithography process. Tumbleston et al. [92] devised this process in order to effectively produce three-dimensional structures out of a liquid bath. In this method, continuous layer-less parts are produced, and relatively faster prints are realizable. Structures with resolutions below 100 microns can also be produced. Additionally, because CLIP is a continuous process, the refresh rate of the projected images does not affect the print speed, thus making the production of smooth 3D objects possible (without any need for model slicing). In CLIP, a continuous sequence of UV images that are generated by a DLP unit is projected through an oxygen-permeable window below a liquid resin bath. The oxygen-inhibited dead zone that is created above this window maintains a liquid interface below the advancing part, while the curing part is drawn out of the resin bath [93]. Haiyang He et al. [94] postulated that a proper continuous elevation speed is key to the success of any continuous printing process and can be very challenging. When the speed is too high, it could result in the newly cured materials not bonding properly to the part. In addition, a very slow speed could result in adhesion between the part and the oxygen-permeable window. Figure 3e illustrates the CLIP process.

### 2.6. Two-Photon Polymerization (2PP)

The development of ultrashort laser systems is creating prospects for very accurate localization of laser energy in time and space [95]. Two-photon polymerization is an additive manufacturing method that uses laser technology to polymerize materials in order to grow the three-dimensional features [96,97,98,99]. When the laser pulses are focused on the photoresist, two-photon absorption is initiated, causing the polymerization to occur. The desired structures are formed inside of the photoresist material, and the regions that are not illuminated are washed away. Two-photon polymerization has better structural resolution and quality than the other stereolithography methods because the laser pulse energy and the number of pulses that are applied can be tuned beyond the diffraction limits [100]. The high-resolution capability of the two-photon polymerization can be harnessed when it is used with an accurate positioning system, such as the piezoelectric stages and/or scanners [100]. Two-photon polymerization has applications in tissue engineering, medical implants, and drug delivery [101,102]. The near IR-laser radiation from this process does not pose any risks to the cells, and it can be used in cell encapsulation and manipulation.

## 3. Additive Manufacturing Processes for Microneedle Manufacturing

The first photopolymerization technique that was used in the fabrication of microneedles was the two-photon polymerization (2PP) process, which employs two-photon absorption in order to initiate polymerization. This stereolithography technique has the capability of printing microneedle arrays with a good printing quality, an excellent resolution, and a high accuracy. In recent studies, 2PP has been adopted in the direct and indirect manufacturing microneedle arrays (through masters or templates). Cordeiro et al. demonstrated how the three-dimensional structures were fabricated using the 2PP technique by manufacturing templates (of different shapes and sizes) and subsequently producing dissolvable and hydrogel-forming microneedles through another microfabrication process called micro-molding [106]. This approach, which was proposed by Cordeiro et al., allows for flexibility in the design of microneedles of different types in an efficient and cost-effective manner. Using the 2PP technique, solid and hollow microneedles were directly printed by Aksit et al. and were subsequently used in the in vitro perforation of a round window membrane. These 2PP-fabricated microneedles showed relatively good quality and sharpness [107]. In a similar manner, the solid and hollow microneedles that were fabricated by Szeto et al. were adopted in the informative sampling of perilymph from guinea pigs [108]. In the works of K. Moussi et al., biocompatible hollow microneedles were directly fabricated using two-photon polymerization; the microstructures that were manufactured using this approach displayed an excellent skin penetrating ability and showed prospects for applications in implantation, transdermal drug delivery, and diagnosis [109]. Faraji Rad et al. also adopted two-photon polymerization in the microfabrication of hollow microneedles. These microneedles were subsequently used in order to generate molds and replicas with very similar structures [110].

Other forms of photopolymerization methods exist for the 3D-printing of microneedles. Instead of the two-photon absorption for the polymerization of materials, laser or light-emission technologies can be adopted in order to achieve the same purpose of polymerization, such as DLP (digital light processing), CLIP (continuous liquid interface production), and laser stereolithography. Amer et al. demonstrated how the micro-molding of biodegradable microneedles can be enhanced by directly printing the molds using the laser stereolithography technique. The anti-cellulite herbal product that was coated in polymeric microneedles was further studied, characterized, and pharmacologically evaluated [111]. Using a similar approach, Krieger et al. 3D-printed microneedle masters that were manufactured and further reproduced into molds using inexpensive and rapid production techniques. These researchers were able to manufacture microneedle molds that were reproducible and customizable [27]. The direct one-step fabrication of 3D-printed cone-shaped microneedles was carried out by Kundu et al. who, through fracture tests, also established that the fracture strength of the fabricated microneedles quadrupled the force of insertion [112]. Microfluidic-enabled hollow microneedles that allow for a heterogeneous mixing and transport of fluids were fabricated by Yeung et al. This study paved the way for the application of hollow microneedles in the delivery of different drug compositions through transdermal routes using one-step laser stereolithography [113]. Additionally, Xenikakis et al. demonstrated the transdermal delivery of model dyes via microchannels that were created on human skin using microneedle patches that were fabricated using an SLA 3D printer. Studies have revealed that human skin that is treated with microneedles has relatively better permeability [114].

**Figure 4:**
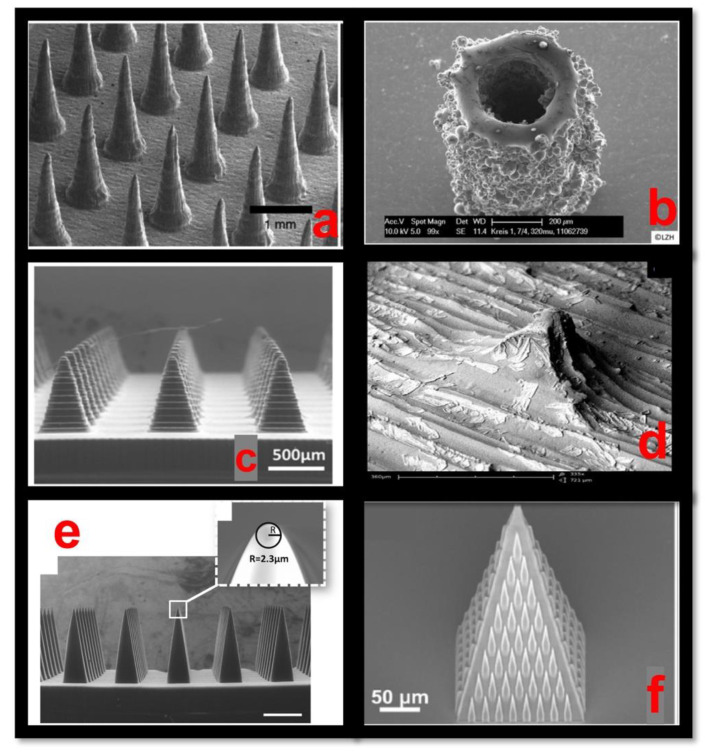
Scanning electron microscopy (SEM) images of microneedles (MNs): (**a**) Stereolithography (SLA) technique MNs [117], (**b**) Selective laser sintering (SLS) technique MNs [115], (**c**) Digital light processing (DLP) technique MNs [117], (**d**) Fused deposition modelling (FDM) technique MNs [118], (**e**) Continuous liquid interface production (CLIP) technique MNs [119], (**f**) Two-photon polymerization (2PP) technique MNs [117].

Microneedles are micrometer-sized needles with a height in the range of 25 to 2000 µm. All of the 3D-printing technologies have the capability to fabricate the microneedles in the above-mentioned range in a reproducible manner with a high resolution and quality. Based on the material and the application type, a particular 3D-printing technology can be utilized. As discussed earlier, microneedles are classified into solid, hollow, coated, dissolvable, and hydrogel-forming microneedles. Table 4 illustrates the different types of 3D-printing technologies based on the microneedle type, the minimum layer resolution, and the application type. The ideal characteristics of the 3D-printed microneedle include the optimum size, the proper mechanical stability, efficient drug delivery, and the ability to remain leak-proof [115,116]. Scanning electron microscopy (SEM) images of the microneedles that were fabricated with the different types of 3D-printing technologies are illustrated in Figure 4.

Some researchers have also made significant efforts towards 3D-print microneedles using the digital light processing (DLP) technique. This technique has an advantage over laser stereolithography printers in that it uses a projected light source to cure all of the sections of each printing layer at once, thereby shortening the time required for printing. Fang et al. presented the manufacturing of 3D-printed dissolvable microneedle patches that were loaded with mesoporous iron oxide nanoraspberry for the treatment of androgenetic alopecia [120]. Johnson and Procopio demonstrated the viability of this technology for the printing of microneedle masters and how the printing outcomes can be improved and optimized by tuning the printing parameters [61]. In another study by Yao et al., customizable resins were used in order to manufacture hydrogel-forming microneedles, with promising results for transdermal applications [121]. Donghyeok Shin and Jinho Hyun studied the 3D-structuring of protein-based microneedles using riboflavin as an enzymatic photo-initiator. The SF microneedles that were manufactured were able to withstand compression forces of up to 300 mN, and they were further applied for the delivery of fluorescent dye molecules into pig skin [122]. The printing quality and the mechanical strength of hollow microneedles were studied using digital light processing (DLP) by Essyrose Mathew et al. The printing quality was optimized by investigating the relationship between the printing angles and the different needle geometries. In addition, it was established that the curing time affects the mechanical strength of the microneedles [123]. CLIP uses the same printing principle as DLP; however, it differs slightly in that it uses an oxygen-permeable membrane that inhibits photopolymerization and creates a dead zone of liquid resin between the advancing structure and the window [117]. Unlike the other stereolithography techniques, the redistribution of the resins at the beginning of a new layer of print is not required, thus ensuring relatively faster and fine printing in CLIP [92]. Table 5 illustrates the prior research work conducted by numerous researchers in the past decade using various additive manufacturing technologies.

One of the most common extrusion-based technologies that is used for microneedle fabrication is fused deposition modeling (FDM). This technology works by heating up the filaments (which are made of thermoplastic materials) to a molten form, which are later extruded through the nozzle in layers in order to produce the final template or object. FDM is not a popular technique for fabricating microneedles because of the poor fidelity and resolution of the printing outcomes. This approach is mostly used in combination with other post-processing techniques in order to improve the quality of the fabricated microneedles. Michael A. Luzuriaga et al. employed the FDM technique to manufacture PLA-based microneedles and improved the quality of the printed microneedles using the chemical etching protocol, realizing the tip sizes up to one micron. The printed microneedle arrays were found to have a good mechanical strength, a high penetrating ability, and the potential for the transdermal delivery of small molecules [26]. Mingxin Wu et al. fabricated microneedles using an extrusion-based approach and adopted a stretching device in order to finetune the top surface of the cylindrical arrays that were formed. These microneedles were subsequently applied for the transdermal delivery of insulin for the treatment of type 1 diabetes [129].

## 4. Applications of 3D-Printed Microneedles

**Figure 5:**
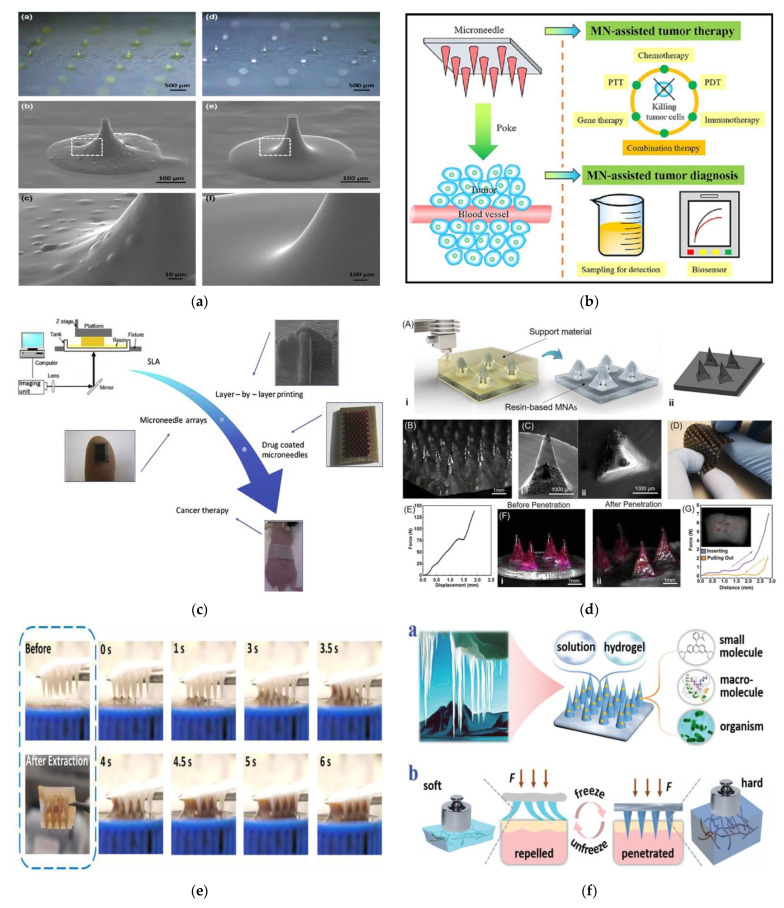
(**a**) Application of microneedles in cosmetics [158], a–d—Stereoscopic microscopic images of dissolving microneedles; e—SEM image of single dissolving microneedles; f—Magnified image of dissolving microneedle surface; (**b**) Application of microneedles in diagnostics and tumor therapy [143], (**c**) Microneedle application in the treatment of diseases [126], (**d**) Transdermal delivery of drugs using 3D-printed microneedle array [159], A—3D-printing showing i—fabrication process. ii—and CAD view of sample MNAs. B—Microscopic images of sample MNAs. C—SEM images of i-front view ii-top view of single hollow microneedle. D—Printing of microneedle arrays for the creation of rigid needles on a flexible base. E—The characterization of mechanical properties of microneedle islands. F—Microscopic view of microneedles array i-before and ii-after penetration into pig skin. G—Characterization of forces(insertion and withdrawal) of microneedles island applied to pig skin; (**e**) Biosensing applications of microneedles [156], (**f**) Application of microneedles in wound healing [160], a—Ice-formation-inspired microneedles made from soft materials (e.g., solution and hydrogels). b—Transition from softness to hardness achieved through freezing.

Microneedles have experienced widespread adoption in areas such as drug and vaccine delivery, cosmetics, therapy, diagnosis, tissue engineering, sample extraction, cancer research, and wound healing (Figure 5).

### 4.1. Drug and Vaccine Delivery

The numerous limitations posed by traditional drug delivery systems [130,131,132,133,134] have led to the development of an alternative approach, using microneedles [135]. In 2021, Cassie Caudill et al. [136] showed that CLIP-fabricated 3D-printed microneedles are a viable option for the enhanced delivery of coated vaccine components (ovalbumin and cytosine phosphoguanine-CpG). The 3D-printed microneedles that were presented by the group were demonstrated to have the potential for enhanced cargo retention in the skin and improved humoral immune response for non-invasive, self-administered vaccination. An additional advantage that is offered by the 3D-printed microneedles in vaccine delivery is that the vaccine-coated microneedles can be transported easily to different locations without any special handling requirements or environmental conditions. In the near future, vaccination rates could improve significantly as patients can self-administer the vaccinations with minimal clinic visits by leveraging the capabilities of 3D-printed microneedles. Limpet-inspired microneedle arrays with high mechanical strength were fabricated using MF-3DP (Magnetic-assisted 3D-printing) by Xiangjia et al. [137] and have been recommended for use in the painless administration of drugs. Sophia N. Economidou et al. [138] developed a device that employed 3D-printing technology of stereolithography for fabricating hollow microneedles and integrated microelectromechanical systems (MEMS). The in vivo testing of the microneedle-based MEMS device showed that it has great potential for personalized transdermal drug delivery.

### 4.2. Cosmetics Applications

Microneedles can transport active cosmetic molecules into the skin by creating microchannels without reaching the nerves [29]. Three-dimensional-printing has been employed for the development of pharmaceutical products for topical skin applications, including skin dressing and products for the transdermal delivery of active ingredients to the skin [139]. Site-based drug delivery can target skin locations such as the stratum corneum, the epidermis, the dermis, the pilosebaceous unit, the hypodermis, and the deeper tissues [140].

It has been reported in the literature by Seng Han Lim et al. [141] that personalized 3D-printed microneedle patches that are fabricated using the digital light processing (DLP) approach can serve as a good alternative for the enhanced delivery of acetyl-hexapeptide 3(AHP-3) for the improvement of skin wrinkles. The resin-based microneedles were formulated from an optimal proportion of polyethylene glycol and vinyl pyrrolidone. In similar research, the geometry of a personalized microneedle patch (PMNP) that was manufactured using the DLP technique was optimized for the delivery of anti-wrinkle peptides [142]. In the treatment of periorbital wrinkles, PMNP has great potential for the transdermal delivery of AHP-3 [142].

### 4.3. Diagnostics and Sample Extraction

With the advancement in science, several tumor diagnosis methods, such as marker detection, endoscopy, and histopathological analysis, have been developed in order to determine the type of tumor and to predict the prognosis [143]. However, some of these methods pose some disadvantages, for example, the procedures that are involved in the marker detection method can be inconvenient and can cause injuries. In addition, the histopathological method can be expensive and invasive to patients. Since microneedles have an excellent skin penetrating ability [144,145,146], they can be used to draw interstitial fluids containing biomarkers, which can be used in order to analyze the patient’s state of health and to potentially detect diseases and tumors. Metal-coated and dissolving microneedles are potentially useful in gene delivery. The literature [147,148,149] has demonstrated how the microneedles can be useful in the delivery of low- and high-molecular-weight agents, including nucleic acids for gene therapy. Microneedling has been identified as one of the four major physical methods that have been developed for the transdermal delivery of therapeutics [119].

### 4.4. Wound Healing

Three-dimensional-printed microneedles were fabricated using visible light dynamic mask micro-stereolithography in combination with pulsed laser deposition utilized for microneedles fabrications using acrylate material with anti-microbial properties. These acrylate-loaded microneedles showed great potential for the treatment of wounds and the removal of skin infections [150]. Microneedle arrays that were filled with drug-eluting hydrogels have been used in the delivery of vascular endothelial growth factor (VEGF), which helps in promoting the epithelization in wounds, as demonstrated by Lindsay Barnum et al. [151]. The permeability properties of VEGF can potentially help in the treatment of non-healing wounds, especially in diabetic patients [152,153]. The mold-free fabrication techniques, such as 3D-printing, have been intensely studied for providing technical support in building complex structures with high drug-loading capabilities and more integrated medical microneedles for wound healing applications.

### 4.5. Biosensing Applications

Three-dimensional-printed microneedles can be used in POC (point-of-care) biosensing applications where the target molecules and biomarkers are easily detectable using the samples that are collected by the microneedle arrays. Microneedle arrays have been described as a good candidate for developing biosensing set-ups [154]. Microneedles are used for sampling, while the detection of the biomarkers takes place in another instrument. The microneedle-based biosensors can provide a pain-free and convenient sampling procedure, followed by minimally invasive techniques for detecting the biomarkers. The microneedle-based biosensor was designed for glucose monitoring, in which the glucose-binding protein was immobilized on nickel-nitriloacetic acid agarose beads [155]. The 3D-printed microneedles with open groove channels for the extraction of liquid samples for biosensing applications were manufactured by Fang Leng et al. [156]. Fang Leng et al. established that the liquid samples containing glucose-based biomarkers can be analyzed in situ using commercial test instruments.

### 4.6. Disease Treatment

Three-dimensional-printed microneedles have been used in the delivery of bioactive agents and therapeutics for the treatment of various forms of disease. Pere et al. fabricated polymeric microneedles using the stereolithography technique and coated the microneedles with insulin using an ink-jetting procedure. The integrity and the stability of the insulin were preserved by the drug carriers, namely trehalose, mannitol, and xylitol [157]. Another piece of literature has reported that 3D-printed microneedles can be adapted for use in anti-cancer skin treatments. Uddin et al. showed the enhanced delivery of cisplatin to A-431 epidermoid skin tumors by photopolymerizing the consecutive layers of the biocompatible resins, followed by the coating of cisplatin formulation on the microneedle surface using inkjet dispensing [126]. Franz cell diffusion and histopathological studies show the rapid rate of cisplatin and the efficacy of cisplatin-loaded microneedles in inhibiting tumor growth. The enhanced delivery of high molecular weight rifampicin using SLA-printed hollow microneedles has also been reported by Vivek Yadav et al. [124].

## 5. Challenges of Microneedle Fabrication

**Figure 6:**
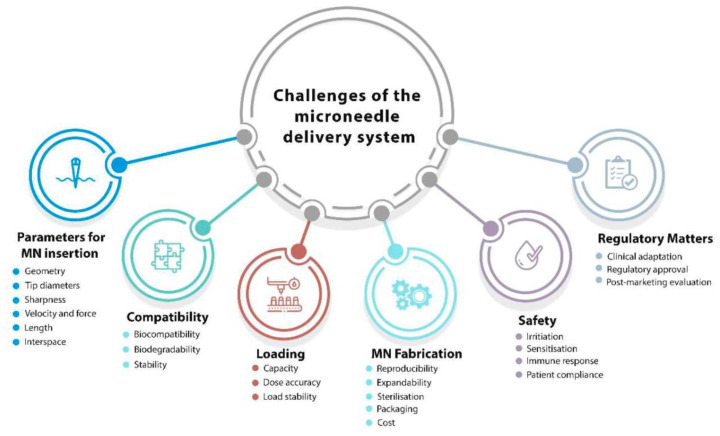
Factors affecting the development of microneedle-based delivery [46].

AM has the potential to fabricate microneedles for multiple applications, as mentioned above; however, it comes with some challenges, which are currently being investigated by various research organizations. The challenges that are involved in the AM fabrication of microneedles include the optimization of its design parameters, biocompatibility, drug loading, safety, regulatory measures, and fabrication viability as summarized in Figure 6 below.

### 5.1. Microneedle Design Parameters

Skin characteristics vary from one person to another, and from one part of the body to another. There are no such microneedle parameters that can be applied or adopted as a standard for everyone. For this reason, the optimization of the microneedle parameters is a paramount topic [161,162,163,164]. Studies have shown that the geometry, the tip diameter, the sharpness, the applied force, the velocity, and the length are of vital consideration when manufacturing microneedles. The geometric structure of the microneedles array affects the mechanical strength and the penetrating ability of the microneedles [46]. It has been demonstrated that square- and triangular-shaped microneedles with sharp edges show a greater capability to penetrate the skin when compared with hexagonal-shaped microneedles. Additionally, microneedles with tip diameters of less than 15 microns are more effective in penetrating the skin than those with larger diameters. Studies have also revealed that the penetration depth of the microneedle arrays varies between 10% and 80% and increases with the application of force and velocity. When microneedles are too tightly packed with little interspacing, the bed-of-nails effect might set in [165].

### 5.2. Compatibility

The biocompatibility of microneedles is essential when they are used in the delivery of drugs [166,167,168]. Biodegradable materials are usually desired because they degrade and can be safely removed from the skin. A critical assessment of the behavior of microneedles under different conditions (such as temperature and humidity) is required, as their stability could have an impact on the loaded therapeutics. Water can alter the mechanical properties of microneedles [169]. Therefore, in order to preserve and prolong their shelf-life (especially the dissolvable microneedles), they must be appropriately stored in a cool and dry place.

### 5.3. Drug Loading

The drug efficiency of microneedles depends strongly on the passive diffusion of the biological formulations in the skin. The administration of large doses of drugs using microneedles is a big challenge, and a larger percentage of drugs could be lost on the skin’s surface [46]. For example, in the delivery of vaccines, dosage variations occur that can reduce the bioavailability of the drug and can reduce the immunological responses of the body. The small surface area of the coated microneedles limits their drug-loading capacity [170], although, a precise quantity of drugs can be delivered.

### 5.4. Microneedle Fabrication

Despite the viability of microneedles for clinical use, the economic implication of manufacturing 3D-printed microneedles for different applications is yet to be fully ascertained. Adhikari, B.B. and Goodson, J.L. et al. [171] gave an estimated cost for the fabrication of microneedles for vaccine delivery. For a population of one million children, the total cost of a vaccination program using a microneedle patch was estimated to be USD 1.5 million compared to USD 2.5 million for the conventional SC injection [171]. Adhikari et al. [171] reported that the cost-effectiveness of microneedle patches depends on several factors, such as the approval rate and the viability of the microneedle patches when compared with the traditional methods. The scalability of microneedle regulatory policies, the clinical adaptation, and the choice of materials and their compatibility plays a vital role in the mass-scale adoption of 3D-printed microneedles.

### 5.5. Safety

The safety assessment of microneedle products is crucial and is carried out during the clinical trials [172]. Skin irritation, sensitization, and immune response, among other metrics, must be evaluated for the safe acceptability and approval for use. One of the reported side effects of using microneedles is erythema [173,174].

### 5.6. Regulatory Measures

The current quality control measures for hypodermic needles may not be suitable for microneedles. A thorough assessment of the risks, stability testing, sterilization testing, and product validation must be performed through the concerned agencies before approval can be secured for the safe use of the products. This validation and approval process could take a long time, which restricts the commercialization of microneedles. According to the FDA regulation regarding the safe use of microneedles (“Regulatory Considerations for Micro-needling Devices, 2017”), microneedles are seen as combination products, and concerns regarding microneedles (as a device) and their therapeutics must be addressed individually.

## 6. Food and Drug Administration (FDA) Guidelines for Microneedle Manufacturing

The FDA has approved the administration of microneedles for patients who are aged 22 years and above. Microneedles are generally considered to be “combination products” [175], therefore, regulatory policies referring to their use consider the micro-needling devices and the safe delivery of the therapeutics that are used with them. The first-ever regulatory policy guiding the safe use of micro-needling products was drafted in 2017. These guidelines were created in order to assist industries in understanding when and when not to classify a microneedle product as a device (in accordance with section 201(h) of the Federal Food, Drug, and Cosmetic Act (FD&C Act), 21 U.S.C. § 321(h). The document also illustrates the regulatory pathway to market micro-needling devices for aesthetic use (“Regulatory Considerations for Microneedling Products-Guidance for Industry and Food and Drug Administration Staff”). The document addresses certain micro-needling products as a term that encompasses instruments with common technological features that include an array of needles, “micro-protrusion” tips, or pins, which can be blunt or sharp, and of varying lengths.

Before microneedles can be adjudged to meet the device definition, two major factors that need to be noted include the firms’ claims and the product design/technological features. Micro-needling products are considered to be a device when they penetrate the living cells of a body, including the dermis and the epidermis (which implies that they are applied in a region that is much deeper than the stratum corneum), and affect the structure or the function of the body. According to the FDA, examples include when microneedles are used in the treatment of scars, wrinkles and deep facial lines, cellulite and stretch marks, dermatoses, acne, and alopecia, and when they stimulate the production of collagen and angiogenesis and promote wound healing. In addition to the firms’ claims and the statements about microneedle products, a microneedle product is classified as a device when its design or technology is such that it impacts the structure or the function of the body. The design characteristics that may be evaluated include the needle length, the needle sharpness, the degree of control of the product over the movement of the needles, and the depth of penetration into the skin.

Microneedles that are not intended for use in the treatment of diseases or other health conditions and do not affect the structure of the body are not considered to be a device (under section 201(h) of the FD&C Act). Microneedles that do not penetrate the human skin and perform the following functions are generally not considered to be a device: facilitate skin exfoliation, improve the skin appearance, give the skin a smoother look and feel, and give the skin a luminous look. The micro-needling devices for aesthetic use are classified as class II devices under 21 CFR 878.4430, are subject to premarket notification (510(k)), and special controls are outlined in the classification regulation.

Micro-needling devices have the potential to cause harm, which is mostly temporary and resolves after a short period following their administration. Micro-needling products might not produce the intended outcomes and could pose risks ranging from skin irritation [176,177], dryness, redness, itching, burning, bruising, and bleeding to less common ailments, such as swollen lymph nodes, infections, hyperpigmentation, and reactivation of herpes cold sores [178] (Micro-needling Devices | FDA).

Below are the FDA recommendations for patients using micro-needling devices [179] (Micro-needling Devices | FDA):

- Patients must consult with health care practitioners who are well trained in the medical procedures that are involved with micro-needling devices;
- Patients must be aware of the common risks that are involved and how microneedles could impact the structure of the body;
- Patients must be aware that they might require more than one procedure in order to obtain the desired cosmetic/aesthetic result;
- Patients should ask questions about the cleaning procedures that are involved in the sterilization of the micro-needling device;
- Patients must be aware that authorized, FDA-approved microneedle products have patient labels.

Micro-needling products are not recommended for persons suffering from ailments such as chronic diabetes, bleeding disorders, hepatitis, and HIV. Patients with active skin infections and those who have (or have had) eczema, psoriasis, vitiligo, actinic keratoses, keloid scars, warts, or birthmarks are also not encouraged to use these devices. Additionally, the procedure may not be suitable for persons who are breastfeeding or those who are currently undergoing chemotherapy or radiotherapy. All medical personnel should carry out a thorough inspection of the micro-needling devices and must confirm that they are listed in the FDA De Novo database or the 510(K) premarket Notification Database. It is highly recommended that only experienced and well-trained practitioners are allowed to carry out or take part in these procedures. Keen attention must also be given to cleaning/disinfection procedures—practitioners are advised not to use micro-needling packages on more than one patient in order to avoid the transfer of infections. All of the medical staff who are involved in the procedure must be aware of the risks that are associated with the use of the product and how to handle complications when they occur (patients should also know about these risks). It is important to report any event of negligence of use immediately to the Food and Drug Administration (FDA).

## 7. Future Prospects

Recent trends in microneedle development have resulted in the fabrication of microneedles from hydrogels. Joseph G. Turner et al. reported that hydrogel-forming microneedles can help in the passive removal of interstitial fluid from the skin, which makes this type of microneedle a viable option for use in biocompatible monitoring or sensing devices. This type of microneedle can provide opportunities for use in self-administered health care monitoring and treatment [180,181]. Microneedles have been widely used in different transdermal applications. Nowadays, dissolvable microneedles are being utilized in the oral delivery of drugs (and macromolecules) throughout the body [182,183,184], and especially in oral devices, in order to target the organs within the gastrointestinal tract. Alex Bramson et al. developed an ingestible capsule called a luminal unfolding microneedle injector, which allows for the oral delivery of drugs into the intestinal tissue using a set of unfolding arms [185]. Regenerative medicine has been evolving at a fast pace, and substantial growth in the investigation of microneedle application in this field will be something to look out for. It is expected that, in the near future, significant improvement can be made in the drug-loading capacity of microneedles and the reproducibility of drug exposure for sustained drug release.

The power of artificial intelligence (AI) (especially machine learning (ML) and deep learning (DL)) can be harnessed and adopted in optimizing the performance parameters and the quality features of 3D-printed microneedle arrays. The tuning of 3D-printing parameters for optimizing the manufacturing of biomedical devices has been reported as a promising field that will further advance the integration of the AI-based prediction of microneedle arrays for the development of advanced healthcare systems, especially in the areas of quality assurance and defect detection in microneedle array features [186]. AI models have shown viability in identifying and matching the similarity metrics between the different microneedle designs in order to predict new and more efficient outcomes for the fabrication of microneedles [186]. In addition, ML has proven to be a suitable approach in the modeling of 3D-printing workflow [187,188,189,190,191]. ML algorithms, in combination with ANN, were able to predict the drug release times of different drug formulations using available data sets, as reported in the literature by B. Muñiz Castro et al. [192]. These ML models can help to develop the field of pharmaceuticals and, more importantly, in the optimization of microneedle array features. The 4D-printing of microneedles is also an emerging prospect. D. Han et al. demonstrated how the 4D-printing methodology was adopted in manufacturing backward-facing barbed microneedles with tips that have relatively better mechanical strength than the barbless microneedle arrays [193]. The microneedles that are fabricated using this approach have better tissue adhesion for a much-improved performance of controlled drug delivery, healing of wounds, biosensing, and soft tissue applications. The 4D-printed microneedles could be a better alternative to painful hypodermic needles in times to come. In the painless administration of COVID-19 vaccines, 3D-printed microneedles can prove to be a good candidate [194,195,196]. In the field of public health, 3D-printed microneedles that were manufactured through CLIP (continuous liquid interface production) were found to enhance the surface coating of the model vaccine components due to the wide surface area that is provided by the CLIP-manufactured microneedles. Three-dimensional-printed microneedles can provide a useful platform for a non-invasive and self-applicable vaccination [136].

## 8. Conclusions

This review article provides a detailed overview of microneedle manufacturing using the 3D-printing technologies by several research groups, including both industry and academia. The key advantages of the additive manufacturing (AM) of microneedles compared to the traditional manufacturing techniques include improved efficacy, safety, therapeutic delivery of drugs, cost-effectiveness, and simplified production. This review provides detailed literature on the different types of microneedles, such as coated microneedles, solid microneedles, hollow microneedles, dissolvable microneedles, and hydrogel-forming microneedles based on the application type. The comprehensive understanding of AM techniques for microneedle fabrication and various application areas, which include cosmetics, drug and vaccine delivery, wound healing, diagnostics, sample extraction, biosensing, and disease treatments are elucidated. This paper also presents the critical challenges, such as the optimization of microneedle design parameters, biocompatibility, drug loading, safety, and regulatory measures, that need to be addressed for the mass-scale adoption of AM techniques for microneedle fabrication. We highlight the regulatory policies, as provided by the FDA, for micro-needling devices with their unique characteristics, including their risks and precautionary measures for their effective implementation. Finally, we discuss recent developments in artificial intelligence and 4D-printing for microneedle manufacturing that hold great promise for self-administration of medications. 

## Figures and Tables

**Table 1:** Advantages and disadvantages of additive manufacturing processes.

Advantages	Disadvantages
Designs can be easily changed, and a range of simple to complex structures can be manufactured.	High-resolution printers are expensive and require huge start-up capital.
Printing parts can be easily optimized—lightweight and heavy objects can be fabricated.	Build speeds can be slow and high-volume production is limited.
Compared to machining, less wastage is generated.	Additional costs might be incurred for the post-processing of finished products for high-quality surface finish.
Parts can be manufactured end-to-end without the need for specialized tools.	3D printers are materials-specific, and this can limit versatility.
Complex geometrical structures can be fabricated without any restrictions.	

**Table 2:** Source: “*Microneedles: A smart approach and increasing potential for transdermal drug delivery system*” [62].

S/N	MICRONEEDLE TYPE	Fabrication Techniques
1.	**Solid Microneedles**	
	*Metal microneedles*	Wet etching Electroplating Laser cutting
	*Silicon microneedles*	3D Laser ablation Silicon dry etching Isotropic etching/anisotropic wet etching
	*Polymer microneedles*	Photolithography
	*Ceramic microneedles*	Sintering lithography Micro-molding
2.	**Hollow microneedles**	Microfabrication Deep X-ray lithography Wet chemical etching Deep reactive ion-etching of silicon Laser micromachining
3.	**Coated microneedles**	Dipping or spraying microneedles with an aqueous solution of increased viscosity to retain formulations during drying Layer-by-layer coating techniques Dipping the microneedles into a coating solution, which is a microwell containing the drug solution
4.	**Dissolvable microneedles**	Micro-molding
5.	**Hydrogel-forming microneedles**	Micro-molding Drawing lithography Injection molding

**Table 3:** An overview of the different types of microneedle material characteristics and benefits [51].

S/N	Microneedle Type	Characteristics	Advantages	Disadvantages	Applications	Material
1	**Solid**	Creates holes in the skin for easy delivery of drugs to the lower layers of the skin	Can be easily fabricated	Prone to infection	Drug delivery and Cosmetics	Silicon Polymer Metal
2	**Hollow**	The drug is filled into the empty space for controlled delivery	Able to handle a large volume of drug solution	Leakage and clogging could occur, and the needle design and insertion methods could pose a challenge	Diagnosis of disease	Silicon
3	**Coated**	Based on its design, it carries a lower amount of drugs	Quick delivery of drugs to the skin	Susceptible to infections	Drug and Vaccine Delivery	Silicon
4	**Dissolving**	Rapid release of macromolecules	Can be easily administered to patients with a one-set application	Takes some time to dissolve, and requires expertise to manufacture	Drug delivery Vaccine delivery Cosmetics	Silicon
5	**Hydrogel-forming**	Absorbs fluids (due to its hydrophilic nature) and swells, creating channels for the delivery of drug molecules	Can be applied to the skin leaving no residues, and has no clogging, unlike hollow microneedles	Potential for localized tissue damage, and has a slow swelling rate	ISF extraction Transdermal drug delivery Disease treatment	Polymer

**Table 4:** Different 3D-printing technologies used for the fabrication of microneedles based on the microneedle type and application [115,116,117].

Technology	Material	Microneedle Type	Minimum Layer Resolution	Power Source	Application
Stereolithography (SLA)	Liquid Polymers	Solid, Hollow, Coated	50–100 µm	UV Light	Drug and anti-cancer agent delivery
Selective Laser Sintering (SLS)	Metals, Polymers, Ceramics, and Thermoplastics	Solid, Biocompatible, Hollow	80 µm	Laser Beam	Drug delivery and disease treatment
Digital Light Processing (DLP)	Epoxides, Acrylates	Solid, Hollow, Coated, Biodegradable, Hydrogel	25–150 µm	UV Light	Skin treatment, wound healing, and drug delivery
Fused Deposition Modelling (FDM)	Thermoplastic Polymers, Metal, and Glass	Solid, Biodegradable	10–100 µm	Heat	Transdermal drug delivery and insulin
Continuous Liquid Interface Production (CLIP)	UV-Curable resins and acrylates	Solid, Coated, Hydrogel, Biodegradable	50–100 µm	UV Light	Drug delivery and diagnostics
Two-Photon Polymerization (2PP)	UV-curable, acrylates, ceramics, and resins.	Solid, Hollow	100 nm–5 µm	UV Light	Drug delivery, sample, and blood extraction.

**Table 5:** Additive manufacturing of microneedles in the past decade.

Reference	3D-printing Method	Microneedle Type	Materials	Proposed Study
Vivek Yadav et al. [124]	Stereolithography (SLA)	Hollow microneedles	Biocompatible Class I resin (Dental SG)	Hydrogel microneedles were fabricated for the transdermal delivery of high molecular weight antibiotics, such as rifampicin (Mw 822.94 g/mol), which suffers from gastric chemical instability, low bioavailability, and severe hepatotoxicity.
Sophia N. Economidou et al. [125]	Stereolithography (SLA)	Coated microneedles	Biocompatible Class I resin, Dental SG	Microneedle arrays were fabricated using a biocompatible resin for transdermal insulin delivery. The 3D-printed arrays were subsequently coated with insulin–sugar films using inkjet printing.
Md Jasim Uddin et al. [126]	Stereolithography (SLA)	Cross-microneedles	Biocompatible photopolymer resin	Novel 3D-printed polymeric microneedle arrays were fabricated for enhanced cisplatin delivery to A-431 epidermoid skin tumors for cancer treatment.
Nesma El-Sayed et al. [127]	Micro Plus High-Res Digital Light Processing (DLP)	Dissolving microneedles	Gold/silver nanoclusters-labeled gelatin nanoparticle	A microneedle master mold was fabricated using a cost-effective 3D-printing technique, resulting in NPs-loaded dissolving microneedles as the final product. A mixture of poly (vinyl alcohol) (PVA) and sucrose was used for the preparation of the microneedle’s matrix. This study provides researchers with the flexibility to develop and analyze new designs and biomaterials for microneedle manufacturing.
Christopher Yeung et al. [113]	Stereolithography (SLA)	Hollow microneedle	Class IIa biocompatible resin (Formlabs, Dental LT Clear)	Hollow microneedles interfaced with microfluidic structures in a single step using SLA techniques were fabricated. This 3D-printed device is particularly applicable to preclinical investigations centered on combinational drug therapy, where the in situ combination of multiple drugs and the tuning of their physicochemical properties lead to more effective outcomes than single or premixed agents alone.
Iakovos Xenikakis et al. [128]	Liquid Crystal Display (LCD) vat Polymerization method	Hollow microneedles	The biocompatible resin used for dentistry applications	Hydrogel microneedles were fabricated for insulin delivery. Different geometries (hexagon, square pyramid, beveled) were 3D printed with a constant height and varying curing time, printing angle, and layer resolution.
Michael A. Luzuriaga et al. [26]	Fused deposition modeling (FDM)	Solid, coated microneedles	Biodegradable polymer	A new method that combines FDM with a post-fabrication etching step to yield ideally sized and shaped needles that can insert, break off, and deliver small molecules into the skin without the need of a master template or a mold.How the natural degradability can be exploited to overcome the key challenge of FDM 3D-printing was reported.
Shaun D. Gittard et al. [100]	Two-Photon Polymerization (2PP)	Solid and Hollow	Ormocer^®^ US-S4 material	In-plane and out-of-plane hollow microneedle arrays were fabricated using the 2PP technique. In addition, the fabrication of microneedles with antimicrobial properties was discussed.
Ashley R. Johnson and Adam T. Procopio et al. [61]	Digital Light Processing (DLP)	Solid microneedles	Autodesk’s Standard Clear PR48 Resin	A commercial desktop 3D printer: Autodesk^®^ Ember™ was utilized for the fabrication of microneedle array patches. The study reports the effect of each type of defect (“stair-stepping”, “aliasing”, and light effects) on the resulting microneedle master structure. The results illustrate that, with the proper use of correction techniques, Autodesk^®^ Ember™ can produce sharp microneedle arrays in less than 30 min per patch.
Ashley R. Johnson et al. [119]	Continuous liquid interface production (CLIP)	Solid microneedles	Poly-acrylic, polycaprolactone, and poly (ethylene glycol)	A novel 3D-printing technique: continuous liquid interface production (CLIP) was utilized for the rapid manufacturing of sharp microneedles with tunable geometries (size, shape, aspect ratio, and spacing). This technology allows for mold-independent, one-step manufacturing of microneedle arrays of virtually any design in less than 10 min per patch. A wide range of materials can be utilized within this platform for encapsulating and controlling the release of therapeutics.
Zhipeng Chen et al. [56]	Magnetorheological drawing lithography (MRDL)	Bio-inspired solid microneedles	Curable magneto-rheological fluid (CMRF)	A novel 3D-printing technique: magnetorheological drawing lithography was utilized for the fabrication of a bioinspired microneedle imitating a honeybee stinger.

## Data Availability

Not applicable.
